# A novel method to identify endotypes and risk factors related to co-occurring obstructive sleep apnea and sleep bruxism

**DOI:** 10.1093/sleep/zsaf238

**Published:** 2025-08-18

**Authors:** Miguel Meira e Cruz

**Affiliations:** Sleep Unit, Centro Cardiovascular da Universidade de Lisboa (CCUL@RISE), Faculty of Medicine of University of Lisbon, Lisbon, Portugal

Sleep bruxism (SB), defined as rhythmic masticatory muscle activity during sleep [1], remains a topic of clinical ambiguity. Historically viewed as a parafunctional behavior or disorder, it is now considered a sleep-related motor activity [2], possibly benign or adaptive in certain individuals. However, the clinical interpretation of SB is complicated by its frequent co-occurrence with obstructive sleep apnea (OSA) [3], insomnia [4], and other systemic conditions [5], highlighting the need for improved phenotyping and risk assessment tools. One particularly relevant intersection is the coexistence of insomnia and OSA [3, 6], which may affect vulnerability to SB and deserves focused clinical consideration.

The recent study by Alkmim de Miranda Diniz et al. offers a novel predictive model for SB in apneic patients [7]. Utilizing six variables, male sex, apnea-hypopnea index, sleep awakenings, total sleep time, antidepressant use, and nonuse of thyroid medications, their nomogram achieved good predictive value (AUC 0.711). This model represents a significant step toward identifying individuals at higher risk for SB, contextualized within the systemic, behavioral, and sleep-related landscape of OSA [7].

Importantly, these findings underscore SB’s multifactorial basis, involving physiological, neurobehavioral, and systemic elements. The predictive model illustrates how SB may reflect not only sleep-related arousals but also broader regulatory frameworks, including arousal instability [8], autonomic modulation [9], and circadian influences [10]. The systemic nature of predictors such as antidepressant use and thyroid dysfunction further supports the notion that SB is integrated into a wider network of physiological regulation.

Recent studies add critical nuance to this perspective. Martynowicz et al. (2025) demonstrated that individuals with low arousal threshold exhibit increased SB intensity, particularly phasic activity, suggesting a compensatory role for SB in terminating respiratory events [11]. Cid-Verdejo et al. (2024) reported that SB frequency decreases with increasing OSA severity, supporting a possible protective function in mild cases of such sleep-related breathing disorder [12]. Meanwhile, Dal Fabbro et al. (2024) emphasized the complexity of managing co-occurring SB and OSA due to limited causality evidence and the influence of patient-specific anatomical and behavioral traits [13].

These findings challenge the traditional view of SB as a disorder and invite a re-evaluation of its clinical significance. SB may exist along a spectrum of motor behaviors, modulated by factors such as arousal threshold, sleep fragmentation, and individual susceptibility [14]. Its relationship with OSA suggests a potential role as a physiological response to airway instability, particularly in individuals with heightened autonomic reactivity [15].

Moreover, the bidirectional relationship between SB and arousals adds complexity. While many SB events are preceded by cortical arousals and sympathetic activation [13], recent physiological research suggests that this pattern is not necessarily universal, even regarding with oromotor activation signaling [16]. This could be particularly relevant in patients with cardiovascular risk, where bruxism-related hemodynamic stress may vary in impact depending on the timing and frequency of events [10].

A critical and underexplored dimension is the temporal patterning of both SB and awake bruxism. Emerging evidence suggests that bruxism episodes may be circadian modulated [10, 17, 18], with activity clusters aligning with peaks in sympathetic tone and cortisol levels. For instance, early morning bruxism (whether awake bruxism or SB) may coincide with increased cardiovascular vulnerability, while evening episodes may impair sleep onset and maintenance, especially in anxious or ruminative individuals.

The neurobiological basis for this circadian modulation is supported by rhythmic regulation of dopaminergic D2 receptors [19] and iron metabolism (eg, serum iron, ferritin, transferrin saturation) [20], both known to influence motor control. These mechanisms are well-recognized in conditions such as restless legs syndrome, a disorder characterized by circadian expression of motor restlessness independent of sleep-wake state [21]. The analogy with restless legs syndrome reinforces the idea that not all bruxism is state-dependent (eg, depending from the awake or the sleep states); some may be governed by internal clocks and external cues, further diversifying its expression and clinical impact.

In this context, two overlapping but distinct pathways of bruxism can be considered: (1) SB as a state-dependent response to sleep-related arousals and respiratory events, and (2) awake bruxism as a circadian-modulated motor activity that may also manifest during the night (nocturnal awake bruxism), potentially mimicking SB. These mechanisms may co-exist in the same patient, leading to diagnostic challenges and variability in clinical outcomes.

From a clinical standpoint, patients often report bruxism at specific times of the day, and preliminary data suggest that the physiological consequences of bruxism episodes vary depending on their timing. For instance, hemodynamic stress linked to SB may be higher during certain circadian phases. Our own research supports this variability, indicating that chronobiological assessment could improve the accuracy of bruxism evaluation and risk stratification [10].

While the nomogram by Alkmim et al. is a cross-sectional tool requiring external validation, it provides a foundation for identifying SB endotypes, especially within the OSA population. The inclusion of systemic and behavioral predictors reflects a shift toward a holistic view of SB, beyond the dental and orofacial framework. Their work supports the growing consensus that SB may be protective, neutral, or pathological, depending on the patient’s phenotype, context, and comorbidities.

Terminology also requires re-evaluation. The label “bruxism” often implies pathology, potentially leading to overdiagnosis and overtreatment. Alternative terms such as “sleep-related masticatory activity” or “masticatory muscle events during sleep” may foster a more neutral and nuanced clinical approach. Furthermore, viewing predictive variables as susceptibility markers (Table 1), rather than risk or protective factors, could refine clinical communication and reduce stigma.

**Table 1:** Clinical Integration of SB Susceptibility Factors in Apneic Patients

Putative susceptibility factor	Clinical implication	Suggested action	Expected outcome(s)
Antidepressant use	Potential SB trigger or confounder	Review medication (eg, anxiety, sleep, pain); assess for COMISA	Reduced SB episodes; improved sleep quality
Nonuse of thyroid meds	Possible subclinical thyroid dysfunction	Recommend thyroid function testing	Identification and management of thyroid imbalance; reduced SB
Sleep awakenings	Fragmented sleep may mimic or trigger SB	Evaluate for insomnia; optimize sleep quality and attenuate arousal low threshold vulnerability	Manage sleep quality; manage heart rate-arousal excitability to lower SB frequency
Elevated AHI	SB may be reactive/protective	Optimize OSA therapy (eg, CPAP, MAD, positional therapy, medication, CBT)	Improved airway patency; potential reduction in SB
Male sex	Increased SB susceptibility	Monitor SB frequency and clinical impact (eg, sleep hygiene, alcohol, etc.)	Early identification of problematic SB; personalized monitoring
Longer total sleep time	Greater opportunity for SB occurrence	Assess need for intervention based on SB impact	Contextualized interpretation of SB impact; avoid overtreatment
COMISA phenotype	Modulates SB arousal-related RMMA expression	Manage insomnia and OSA. Improve arousal susceptibility in an integrated manner (eg, sleep hygiene, medication) in collaboration with psychologist and physician	Improved sleep stability; possible SB reduction
Circadian timing of SB	May influence cardiovascular risk or sleep disruption	Assess SB timing; assess if AB is also present; consider chronotherapeutic strategies	Minimized risk during vulnerable times; optimized treatment timing
Autonomic activation	Potential cardiovascular strain in vulnerable individuals	Monitor BP and HRV; assess cardiometabolic risk	Reduced cardiovascular stress; improved patient safety
Ambiguous SB-arousal chronology	Influences interpretation of SB’s role in sleep disruption	Tailor management based on patient-specific patterns	Targeted interventions; improved understanding of SB dynamics
Terminology (“bruxism”)	May lead to overdiagnosis and overtreatment	Use neutral language; contextualize clinical significance	Improve sleep medicine dentist and physician interdisciplinary collaboration; enhanced patient-clinician communication

This table summarizes putative key susceptibility factors influencing the occurrence or expression of SB in patients with OSA, based on current evidence and clinical experience. For each factor, the potential clinical implications, suggested clinical actions, and expected outcome(s) are outlined to guide personalized assessment and management strategies. The table reflects a critical reappraisal of aspects supported by the results of the article by Alkmim de Miranda Diniz et al. (2025) incorporating broader conceptual considerations that require external validation in future studies.

Importantly, predicting SB should not mandate intervention. Rather, prediction should guide context-sensitive and personalized care. In patients with dental damage, orofacial pain, or cardiovascular vulnerability, monitoring and intervention may be warranted. In others, SB may be benign or even beneficial, particularly if it plays a role in stabilizing the airway in OSA.

Future research should aim to refine phenotyping and predictive models through longitudinal studies, incorporating 24-h EMG, PSG recording during daytime sleep/nap monitoring, arousal threshold profiling, and circadian variables. Such integrative approaches can help determine when SB is clinically significant and which patients may benefit from intervention. Additionally, the role of environmental, behavioral, and genetic factors should be explored to further delineate SB endotypes and optimize management.

In conclusion, the study by Alkmim et al. provides a novel method to identify endotypes and susceptibility markers related to SB and its interaction with OSA. Their model adds to a growing body of evidence suggesting that SB is a heterogeneous phenomenon, shaped by systemic, behavioral, and temporal dynamics. Rather than ask whether SB is a disorder, a marker, or an epiphenomenon of normal sleep, we should ask when, why, and for whom it matters and under what physiological and clinical conditions it warrants attention.
